# Canine Sense and Sensibility: Tipping Points and Response Latency Variability as an Optimism Index in a Canine Judgement Bias Assessment

**DOI:** 10.1371/journal.pone.0107794

**Published:** 2014-09-17

**Authors:** Melissa J. Starling, Nicholas Branson, Denis Cody, Timothy R. Starling, Paul D. McGreevy

**Affiliations:** 1 Faculty of Veterinary Science, University of Sydney, Camperdown, New South Wales, Australia; 2 Deakin Research, Deakin University, Burwood, Victoria, Australia; 3 Indice Ecotech Pty Ltd, Camberwell, Victoria, Australia; 4 Wikimedia Foundation Inc., San Francisco, California, United States of America; CNR, Italy

## Abstract

Recent advances in animal welfare science used judgement bias, a type of cognitive bias, as a means to objectively measure an animal's affective state. It is postulated that animals showing heightened expectation of positive outcomes may be categorised optimistic, while those showing heightened expectations of negative outcomes may be considered pessimistic. This study pioneers the use of a portable, automated apparatus to train and test the judgement bias of dogs. Dogs were trained in a discrimination task in which they learned to touch a target after a tone associated with a lactose-free milk reward and abstain from touching the target after a tone associated with water. Their judgement bias was then probed by presenting tones between those learned in the discrimination task and measuring their latency to respond by touching the target. A Cox's Proportional Hazards model was used to analyse censored response latency data. Dog and Cue both had a highly significant effect on latency and risk of touching a target. This indicates that judgement bias both exists in dogs and differs between dogs. Test number also had a significant effect, indicating that dogs were less likely to touch the target over successive tests. Detailed examination of the response latencies revealed tipping points where average latency increased by 100% or more, giving an indication of where dogs began to treat ambiguous cues as predicting more negative outcomes than positive ones. Variability scores were calculated to provide an index of optimism using average latency and standard deviation at cues after the tipping point. The use of a mathematical approach to assessing judgement bias data in animal studies offers a more detailed interpretation than traditional statistical analyses. This study provides proof of concept for the use of an automated apparatus for measuring cognitive bias in dogs.

## Introduction

Animal welfare science focuses on the assessment and the potential optimisation of the quality of life of animals. Animal welfare studies have traditionally focused on identifying negative states tied to stressors such as those causing pain, fear, anxiety and frustration [1], [2], as it was assumed that these conditions reflect poor welfare and that therefore good welfare results from the absence of these states [1], [2]. However, there are problems with this approach. For example, negative states are adaptive and consequences of a stress response may be protective [3]. It has been suggested that assessments of animal welfare should not focus purely on avoiding pain and suffering, but should also place value on positive, pleasurable activities and resources [4]. It is therefore of growing importance to identify accurate indicators of positive and negative affective state in animals.

One potential method of identifying positive and negative affective states in animals is testing cognitive bias. Cognitive bias is a term that has been used in the human literature to describe the effects of affective state on a range of cognitive processes such as information processing and decision-making [5], [6]. It is now being put to similar use in non-human animals, where it has been found that the cognitive process of judging how to interpret ambiguous signals is under the influence of current affective state. This specific form of cognitive bias is called judgement bias. A judgement bias refers to how animals interpret ambiguous signals and whether they expect more positive or negative outcomes. A negative affective state leads to an expectation of negative outcomes and thus a negative bias in the interpretation of ambiguous signals. This has been referred to in the animal literature as pessimism [7], [8]. In contrast, a positive affective state leads to an expectation of positive outcomes and positive biases in signal interpretation, which has been referred to as optimism [9], [10]. Environmental conditions that induce either a state of positive or negative affect can be used to test this concept in animals by changing environmental conditions to induce either a putative positive or negative affect and then testing whether judgement bias changes correspondingly. This approach has been reported in rats [11], [12], starlings [7], [9], [10], [13], [14], sheep [15]–[17], chickens [18], [19], cats [20], primates [21], [22], pigs [14], dogs [23], [24] and honeybees [25]. In the species studied to date, negative judgement biases positively correlate with conditions known to induce negative affect, and positive judgement biases positively correlate with conditions known to induce positive affect. Furthermore, pessimism has been reduced with the use of drugs designed to reduce fear in lambs [17] and pessimism has been associated with physiological indicators of elevated distress in honeybees [25]. Complexities in optimism and pessimism expression have been recorded in starlings [13] and tufted capuchins [22], in that higher frequency of stereotypic behaviours have been associated with heightened pessimism. Similarly, dogs that show indications of heightened separation-related distress have been shown to be more pessimistic than those with fewer indicators of separation-related distress [23]. These results support the use of judgement bias in animals as a potential indicator of both positive and negative affective state, but the role of personality in the expression of optimism and pessimism remains unclear.

This study provides proof of concept for the use of a novel, portable, automated apparatus to train an operant, auditory discrimination task and subsequently test cognitive bias. The apparatus auto-shapes dogs to perform an auditory discrimination task, then records their latency to respond to reveal their expectations and therefore their judgement bias. It was designed to collect data on judgement bias in a range of dogs from different environments, investigate population levels of optimism and pessimism and explore factors that may affect the expression of judgement bias. This study reports on baseline optimism in companion dogs, dogs in training for assistance roles, and security and detection dogs, and introduces a novel method of analysing cognitive bias data to produce an optimism index.

## Methods

### Ethics Statement

The protocols used in this study were approved by the Animal Ethics Committee of the University of Sydney (Approval no: 20101111 5407). Written consent was obtained from the dogs' carers prior to the commencement of the study.

### Subjects

The subjects included 40 dogs of various breeds. Seventeen of the dogs (aged 1–6 years) were recruited via a positive training and pet boarding company based in the North Shore suburbs of Sydney, Australia. These dogs belonged to companion animal owners and thus were subject to variable housing, feeding and exercise arrangements. Twelve dogs were sourced from Assistance Dogs Australia's (Heathcote, NSW, Australia) advanced training facility. These dogs were 1–2 years old. Eleven dogs (aged 1–3 years) were sourced from a private security company. Dogs were recruited from different environments chiefly in the interests of accessing as many dogs as possible. Details of the dogs in the study are shown in **Table 1**. Dogs older than eight years were excluded to avoid recruiting dogs that may have been affected by canine cognitive dysfunction. Dogs younger than one year were excluded to avoid the possible influence of social immaturity on cognitive bias.

**Table 1:** A history of dogs in the study, showing their source (ADA = Assistance Dogs Australia), breed, sex (M = male, F = female) and reproductive status (N = neutered, E = entire), the protocol they were assigned to (A = milk tone lowest, B = milk tone highest), the side the milk was dispensed to, the training phase reached before the dog was excluded, and the reason for exclusion.

Dog	Source	Breed	Sex/Reproductive status	Protocol	Milk tray side	Phase reached	Reason for exclusion
Jazz	Public	Spoodle	F/N	A	R	TP1	Inconsistency in targeting rate, ear interference
Murphy	Public	Whippet×Border collie	M/N	A	R	TP1	Rate of targeting too low
Declan	Public	Labrador retriever	M/N	A	L	CBT	
Ellie	Public	Labrador retriever	M/N	A	R	CBT	
Oscar	Public	Schnauzer	M/N (implant)	B	R	TP1	Avoided lactose-free milk
Jack	Public	Australian cattle dog	M/N	B	R	CBT	
Zack	Public	Maltese cross	M/N	A	R	TP2A	Targeting extinguished
Apollo	Public	German shepherd dog	M/N	A	L	Habituation	Avoided lactose-free milk
Ellie U	Public	Groodle	F/N	A	R	TP3	Targeting extinguished
Abbie	Public	Golden retriever	F/N	B	L	CBT	
Diesel	Public	Groodle	M/N	A	L	TP3	Targeting extinguished
Sinbad	Public	Border collie	M/N	A	R	TP3	Targeting extinguished
Jenna	Public	Border collie	F/N	A	L	CBT	
Jesse	Public	Border collie	F/N	A	R	CBT	
Lola	Public	Labrador retriever	F/N	B	L	CBT	
Diesel T	Public	Rhodesian ridgeback	M/N	B	R	CBT	
Archie	Public	Pug x Schnauzer	M/N	B	R	CBT	
Chance	ADA	Labrador mix	M/N	A	R	CBT	
Hudson	ADA	Labrador retriever	M/N	B	L	CBT	
Jaxon	ADA	Labrador retriever	M/N	B	R	CBT	
Biscuit	ADA	Labrador retriever	M/E	B	R	CBT	
Risky	ADA	Labrador mix	M/N	B	L	CBT	
Willow	ADA	Golden retriever	F/N	B	R	CBT	
Colonel	ADA	Golden retriever	M/N	B	L	CBT	
Penfold	ADA	Golden retriever	M/N	A	L	TP3	Left facility
Paws	ADA	Labrador retriever	M/N	B	R	TP3	Targeting extinguished
Tila	Security	German shepherd dog	F/E	B	L	TP1	Rate of targeting too low
BJ	Security	German shepherd dog	F/E	A	R	TP1	Rate of targeting too low
Arnie	Security	German shepherd dog	M/E	A	L	CBT	
Kaiser	Security	German shepherd dog	M/E	A	L	TP2	Targeting extinguished
Shadow	Security	German shepherd dog	F/E	B	R	TP1	Rate of targeting too low
King	Security	German shepherd dog	M/E	B	L	Habituation	Avoided apparatus
Jessy	Security	English springer spaniel	F/E	B	R	CBT	
Panda	Security	English springer spaniel	F/E	A	R	CBT	
Ruby	Security	German shepherd dog	F/E	A	L	TP3	Targeting extinguished
Zena	Security	German shepherd dog	F/E	B	R	TP2	Targeting extinguished
Kato	Security	Belgian Malinois	M/E	B	L	TP2	Rate of targeting too low
Chilli	Public	Belgian Malinois	F/N	A	L	TP1	Rate of targeting too low
Krash	Public	Belgian Malinois	M/N	B	L	TP3	Targeting extinguished
Nellie	ADA	Labrador retriever	F/N	B	R	CBT	
Ronnie	ADA	Golden retriever	M/N	A	R	TP3	Targeting extinguished
Buster	ADA	Greyhound	M/N	B	L	TP3	Failed to learn discrimination

**No dogs that reached CBT (cognitive bias tests) were excluded.**

### Apparatus

**Figure 1:**
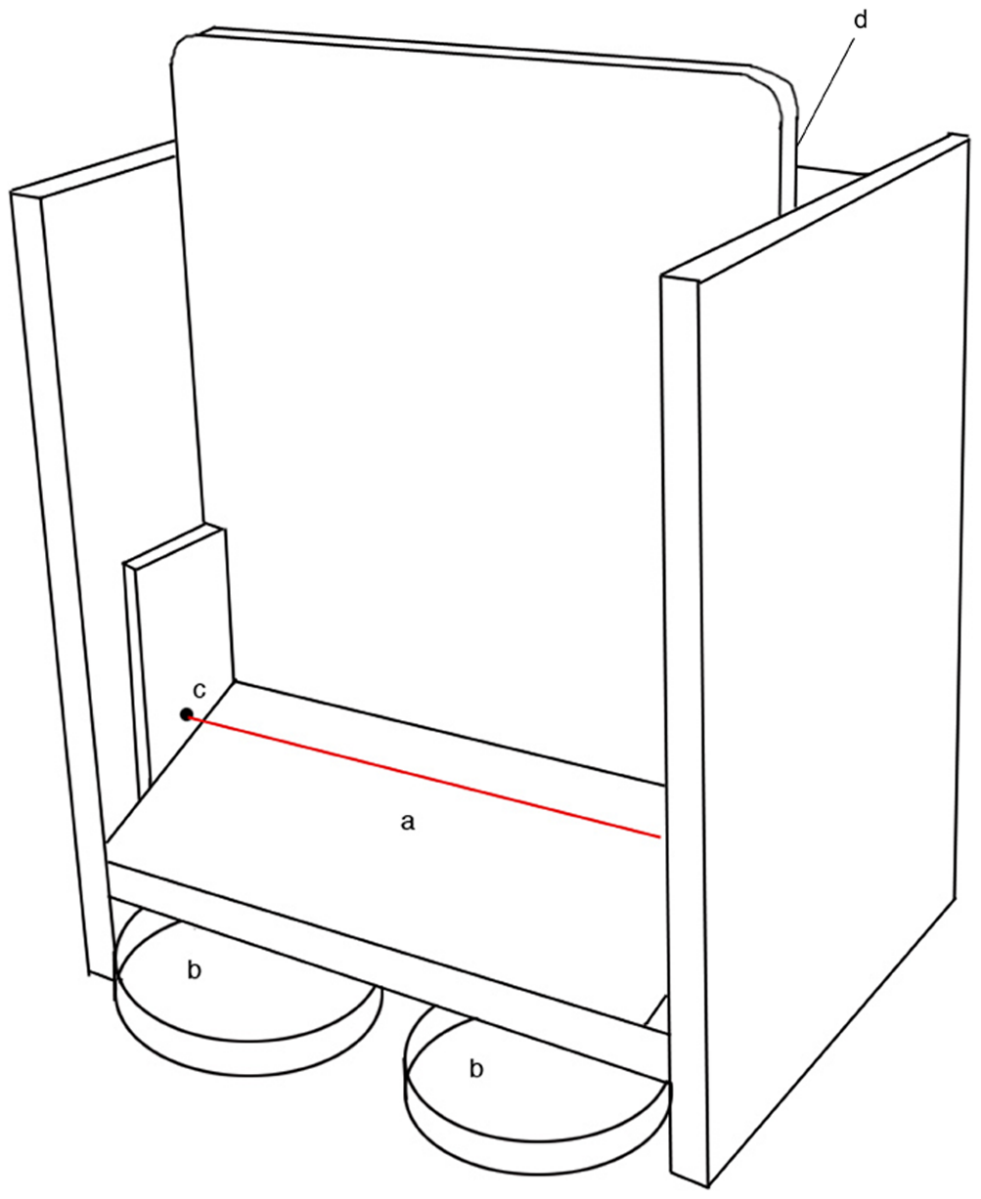
A diagram of the apparatus used in the study. a = target, b = milk and water trays, c = photointerruptor shown with a red line, d = LCD screen and controls (not visible). Trays are stowed under the target zone for ease of transport, but are moved out in front of the target when in use.

The apparatus used in this study was designed to be portable and easy to set up and operate. A diagram of the apparatus is shown in **Figure 1**. It consisted of three major external components: an interactive target that detected movement through the use of an infrared photointerruptor, and two feed trays assigned to either lactose-free milk or water. As a diet high in lactose is associated with diarrhoea in some dogs [26], lactose-free milk was chosen as a liquid reward to avoid causing digestive upsets. Throughout training and testing, dogs received a set volume of lactose-free milk and water ranging from 1–5 mL, depending on their bodyweight.

The apparatus prototype was constructed around an Arduino Uno micro-controller board (SmartProjects, Italy). The Arduino Uno controlled an LCD screen (V1.2 and V1.2: DFRobot, Beijing, China; V2.1: FORDATA ELECTRONIC Co. LTD, China), two peristaltic pumps (SmallPumps, Arlington, Texas, USA; part # SP200 517), six pin buttons (generic manufacturer, part# SP0710) used to set the training program variables, a power switch (generic manufacturer, part #:SK0960), and an infrared photointerruptor. The photointerruptor consisted of an infrared LED (Osram, Malaysia) and a phototransistor (Vishay, Germany). The flow rate on the pumps was approximately 100 mL/minute. Peristaltic pumps deliver small amounts of liquid by compressing a silicone delivery tube, thus ensuring the tubes were primed to deliver liquid the moment the pump was activated. The pumps were calibrated by measuring the volume of liquid they dispensed in a second. Reservoirs in the form of 500 mL intravenous transfusion bags were connected to plastic and silicone tubing, which delivered milk and water to the two pumps. Plastic tubing also delivered liquid from the pumps to two feed trays fixed in front of the target. Each delivery tube was dedicated to delivering either milk or water, and could be configured to deliver fluid into either the left hand tray or the right hand tray, thus allowing milk to be delivered to either side and controlling for any individual's bias to prefer one side over the other. Two alternate auditory protocols were generated to account for tone-generated biases. Protocol A used the lowest tone as the milk tone and the highest as the water tone, and this was reversed in Protocol B.

Four buttons provided a means to select options displayed on the LCD screen. This interface allowed the operator to select the weight class of the dog (0–7 kg, 8–27 kg, 28–47 kg, 48 kg+), the auditory protocol, the training phase, and to start the training session. The remaining two buttons activated the two pumps outside of the training program. This was essential for cleaning the tubes and pumps and priming the tubes before the training program began. A speaker volume control dial allowed adjustment of the volume of the tones emitted. The frequencies of auditory tones are shown in **Table 2**.

**Table 2:** Frequency (Hz) of auditory tones used in training and testing.

Protocol A	Protocol B	Frequency (Hz)
Milk	Water	523
P1	P9	600
P2	P8	690
P3	P7	792
P4	P6	910
P5	P5	1046
P6	P4	1201
P7	P3	1380
P8	P2	1585
P9	P1	1821
Water	Milk	2092

Protocol A is the reverse of Protocol B to control for possible selective attention effects that may influence response latencies.

Milk and water tones were used in training and testing and the nine probe tones (P1–P9) in testing only.

### Habituation

Two coin tosses were used to assign each dog randomly to an auditory protocol and a milk tray side. Dogs were then habituated to the apparatus through a brief habituation program that involved placing a set number (n = 14) of small liver treats around the apparatus for the dogs to find and consume. The apparatus was turned on and set to Training Phase 1 (TP1). The tone volume was increased in successive triggering events until the dog's ears came up and forward when the marker tone sounded. At this point, the volume above background noise of the tone was recorded (in decibels) using a sound level meter held within 5 cm of the apparatus speaker, and the apparatus volume was set at this level above background noise prior to all interactions the dog had with the apparatus. If dogs did not show an observable response to the tone, the volume was set at maximum for that dog. The milk pump was activated manually when the dog was investigating the apparatus. The dog was allowed to consume the milk delivered to the milk tray and the milk pump was activated manually until the dog moved away from the milk tray or until the pump had run for approximately 7 seconds if the dog did not move away. Any dog that did move away was given approximately 5 seconds to return to the milk tray. If they did not return on their own, dogs were encouraged with verbal coaxing and tapping of the milk tray by the experimenter. This process was repeated until each dog had consumed milk from the tray without a reaction to the sound of the pump for approximately 7 seconds.

### Training and Judgement Bias Testing

Dogs were trained in a go/no-go discrimination task where they were required to touch a target with their nose after a tone in order to trigger the delivery of a lactose-free milk reward or water. The tone informed the dog which outcome would be delivered, and thus whether they should go ahead and touch the target or avoid touching. When dogs showed a significant difference in their response to the two tones, the dog's judgement bias was assessed by presenting 9 new, ambiguous tones that fell between the milk and water tones.

**Figure 2:**
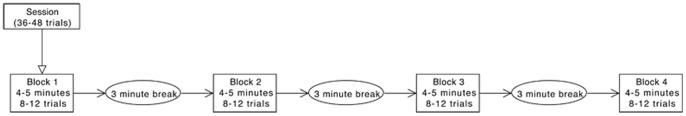
A visual representation of training and testing session structure. Each session has a total of 32–48 tone presentations, depending on the phase (see Table 3). The tones are presented in 4 blocks, with a 3-minute rest period between blocks.

Three training phases were used to train the dogs in the discrimination task. These phases and criteria for learning are summarised in **Table 3**. The testing phase was the judgement bias test itself and was the only phase that included ambiguous signals. Training and test sessions lasted no longer than 30-minutes and consisted of four 5-minute training blocks and a 3-minute rest period between each training block. The structure of training and test sessions is shown in **Figure 2**. Dogs that had not met success criteria within 30-minutes, were given a subsequent training session within 24 hours. Dogs received up to two sessions a day and had no more than five days between sessions.

**Table 3:** Summary of training phases and cognitive bias testing phase.

Phase	Training Objective	Structure	Criterion
TP1	Dogs to move nose through photointerruptor beam towards visual target.	Dogs may receive a maximum of one reward every 8 seconds (s).	Reward trigger rate of at least 8 in 2 of 3 training blocks
TP2	Dogs to move their nose to the target on cue.	Milk tone played, 10 s window to respond, 20 s Inter-trial Interval (ITI).	Touches target at least 80% of time after tone for 2 of 3 training blocks
TP2A	Reduce reinforcement rate	Milk tone played, 10 s window to respond, 30 s ITI.	Touches target at least 80% of time after tone for 2 of 3 training blocks
TP3	Dogs to discriminate between 2 tones.	Milk or water tone played pseudo-randomly, 10 s window to respond, 20 s ITI.	Milk latency significantly shorter than water latency (Mann-Whitney U-test)
CBT	Test cognitive bias	2×9 probes, 15 water, 15 milk presented pseudo-randomly, 20 s ITI.	N/A

The experimenter was always within 3 m of the apparatus and always in sight of the dog during training and testing. The experimenter could hear the tones, but was able to predict the tone that would be presented only when the previous two tones had been the same. Given most of the dogs worked or lived with humans, it was challenging to control and impossible to eliminate experimenter effects on dog behaviour while still being within sight of the dog and monitoring their interaction with the apparatus. However, experimenter intervention followed a protocol in an attempt to control such effects. If the dogs did not respond to two milk tones in a row during training phases, the experimenter called their name once and pointed to the apparatus. If the dogs did not approach, this was followed by calling “come here” in a light, high tone and clicking the fingers. If the dogs still did not approach, this procedure was followed after dogs had failed to respond to a further two milk tones. If the dogs still did not approach, the procedure was repeated after the dogs had failed to respond to a further 4 milk tones. If the dogs did not respond to any further tones in that block, the session was aborted at the end of the block. If, during training, the dogs lay down too far from the apparatus to access the target and did not get up upon hearing one tone, the apparatus was moved to within 30 cm of the point where their chest touched the ground.

#### Training Phase One (TP1)

TP1 trained dogs to touch the target by delivering a reward each time the dog passed through the photointerruptor in front of the target. There was an 8-second block on the photointerruptor after it had been activated so that subsequent triggering did not result in the immediate delivery of further rewards. This prevented the delivery of a double dose of lactose-free milk before the dog had consumed the first reward. There was no set number of trials in this phase, as no tones were presented and dogs would receive a reward any time they touched the target outside of the 8-second block after a previous touch. The maximum number of trials the program could support in a session of this phase with the 8-second block was 150 and the minimum was 0. Dogs were given at least one full session, after which the criterion in **Table 3** was implemented if it had not already been met.

#### Training Phase Two (TP2)

TP2 trained dogs to move their nose to the target on cue. The cue was an auditory tone (henceforth “milk tone”). The training protocol is shown in **Table 3**. Dogs were given one full session on TP2, after which criterion in **Table 3** was implemented if it had not already been met. There were 48 trials in a session. Dogs were excluded from the study if they were not able to meet the criterion for success in three sessions.

#### Training Phase 2A (TP2A)

The objective of TP2A was to ensure dogs were responding to the tone and not the fixed interval between tones, and to gradually ease dogs into the lower reward rate of TP3 and cognitive bias tests. Criterion in **Table 3** was implemented. There were 32 trials in a session. Dogs were excluded from the study if they were not able to meet the criterion for success in three sessions.

#### Training Phase Three (TP3)

The objective was to train dogs to discriminate between the milk tone and a new tone (“water tone”) that signalled that moving the nose to the target would result in the delivery of water instead of milk. Milk and water tones were played such that no more than two of the same tones were played in succession. This was in alignment with other similar cognitive bias studies in animals [13], [15]. Tones were followed by a 10-second response window, reward delivery if applicable, 20-second inter-trial interval (ITI), and then the next tone. The criterion for success in TP3 was that dogs demonstrated their discrimination between milk and water tones by touching the target significantly faster after milk tones than after water tones. This was determined by a one-tailed Mann-Whitney U test. Dogs were required to show this discrimination in two successive training sessions or two out of three training sessions. They were given a maximum of 25 sessions (48 cues per session) on TP3 to achieve the criterion.

#### Cognitive Bias Test (CBT)

Cognitive bias testing involved the presentation of auditory probes. The apparatus logged the latency of the dog to respond to probe tones by automatically recording when the dog broke the infrared beam of the photointerruptor. The probes were interspersed throughout a regular training session. Probe tones were presented randomly and milk and water tones were presented pseudo-randomly, with no more than two milk tones or two water tones in a row. Each of the 9 probes were presented twice and milk and water tones were each presented 15 times throughout the test. Each dog was given 3 cognitive bias tests over the space of 2 weeks. These were alternated with two regular training sessions of TP3 in the sequence CBT1→TP3→CBT2→TP3→CBT3 to ensure responses to milk and water tones remained consistent.

### Statistics

All statistical analyses were carried out in R, version 2.15 (R Foundation for Statistical Computing). A one-tailed Mann-Whitney U-test (with a significance level of p<0.05) was used on each dog in TP3 to test whether dogs were significantly faster to touch the target after milk tones than after water tones. A one-tailed test was used because, for the judgement bias test to be meaningful, the average latency for milk tones had to be significantly less than the average latency for water tones rather than significantly different in either direction. Davis et al. [27] have shown the startle reflex to be sub-cortical and to not involve cognitive processing. As such, a minimum response latency of 500 ms was set to exclude responses unlikely to be cognitive. This was based on the minimum response time to auditory cues in rats [28]. Such responses were substituted with the mean latency for the corresponding tone in that session if the response was to milk or water tones. If latency was less than 500 ms for a probe tone, that response was excluded as there were far fewer responses available to form an accurate mean substitution, and much greater variability in probe responses. The ‘survival’ package was used to analyse cognitive bias tests using a Cox proportional hazards regression model. This model was chosen as the data were censored at 10 seconds. If dogs had not touched the target within 10 seconds of the tone, their latency was recorded as 10 seconds and marked as censored. The dependent variable in a survival model has two parts: the event indicator and the latency to the event. In this case, the event indicator is touching the target (or reaching the end of the 10-second window without touching the target), and critical latency is the time it takes to touch the target after a tone. The regression model was built using the stepwise method. The terms in the model were tested using the ‘anova’ function, comparing the model containing the new term with a model excluding the new term and retaining the term if there was a significant difference in models. Terms that were considered for inclusion were “Dog”, “Trial” (CBT1-3), “Protocol” (A or B), “Background” and “Cue” was the dependent variable.

The results of cognitive bias tests were processed in Mathematica 8 (Wolfram Industries) and interpreted in terms of a mathematical model rather than a frequentist statistical model. This was done to enable us to identify the clear but subtle patterns in the results without depending on measures of statistical significance that may not be appropriate for use with a small sample size such as that reported here. The mathematical model can be defined in words and is shown in **Table 4**. This is based on simple statistics. A consistent pattern was detected from the data of the 20 dogs that completed cognitive bias tests whereby average latencies for each tone suddenly increased by 100% or more. This was defined as the tipping point. The tipping point was used to indicate where expectation of probe outcomes switched from positive to negative. A variability score was calculated from data following the tipping point (excluding data related to water tones) to give a measure of how quickly dogs responded to probe tones after the expectation switch. The variability score was simply the sum of the average latency at each tone divided by the standard deviation of latency at that tone, i.e. average latency at tipping point/SD at tipping point+average latency at probe adjacent to tipping point/SD at that probe +… through to the probe adjacent to the water tone. If a tipping point were at P7, the formula would be: (average latency at P7/SD at P7)+(average latency at P8/SD at P8)+(average latency at P9/SD at P9). The purpose of this was to devise a measure of how variable responses were after the tipping point. High variability would indicate dogs that are still in a state of flux with their interpretations of probes, but are still responding to some tones relatively quickly as if they were expecting a positive outcome.

**Table 4:** A description of the mathematical model used to interpret latencies.

Component	Definition	Interpretation
Tipping point	The probe number where average latency to respond increases by 100% or more from the average latency of the previous tone in a data table sorted by tone frequency from milk to water.	This provides an indication that the dog has discriminated between tones. Scored by which tone first large increase in latency occurs (1–11, where 1 = milk tone and 11 = water tone).
Null response	A measure of the proportion of responses with latencies shorter than the average latency for all dogs.	Can be compared to average latency to show whether the difference between the dog's average response and the proportion of faster than average responses is positive or negative.
Variance score	Fluctuation in average latencies after the tipping point measured by standard deviation/average latency for that tone. This calculation is summed for all tones from the tipping point to give a variability score.	High variability score indicates both quick response and very slow or no response, characteristic of optimistic dogs that either respond fast or not at all. Variation remains low in more pessimistic dogs indicating low levels of response and slow responses.

The results from the mathematical model were compared with subjective rankings of the dogs derived from the owners or trainers. Three dog ‘types’ were described in subjective terms based on the response latency data and behavioural data recorded during training and testing. These descriptions are shown in **Table 5**. Descriptions were sent to two separate people who knew the dogs well – either living with them or training them. These people were asked to categorise the dogs according to the type that best described them. Categorising dogs as between types was allowed.

**Table 5:** Subjective descriptions of the behaviour of dogs during training and testing.

Optimism rank	Type	Description of dog's behaviour
1	1	Dog responds more quickly to signals than other dogs, but may do the ‘wrong’ thing. The dog may not be bothered by an incorrect response or may appear frustrated, but will usually eagerly try again without needing very much encouragement. Dog does not tend to give up easily.
2	1–2	No description given.
3	2	Dog responds neither quickly nor slowly to signals and does the right thing on average. When the dog gets something wrong, it may appear disappointed or discouraged, but it will try again with a little coaxing or encouragement.
4	2–3	No description given.
5	3	Dog may prefer not to risk incorrect responses, responding slowly or not at all to signals unless very familiar with the correct response. When the dog gets something wrong, it may appear distressed or be difficult to coax into trying again, or may simply wait passively for a signal it knows.

Owners and trainers were asked to place dogs in one of the response categories described, but were allowed to place them between categories to reflect the continuous nature of the descriptions. Dogs were categorised according to their empirical variability scores (optimism rank), giving an indication of how variability scores might relate to how owners and trainers subjectively viewed the dogs' behaviour.

## Results

The fate of all dogs in the study is shown in **Table 1**. Twenty of the 40 dogs included in the study completed all three cognitive bias tests. The exclusion rate was highest in security dogs (72%, n = 11), lower in pet dogs (47%, n = 19) and lowest in Assistance Dogs Australia advanced training dogs (33%, n = 12). Reasons for exclusion of dogs during the training program included inconsistent or low rates of targeting resulting in a failure to meet the criterion for TP1 and extinction of targeting in later training phases when reinforcement rates decreased. In addition, two dogs appeared to dislike the lactose-free milk, avoiding the milk tray and ignoring attempts to coax them towards it. Dogs that completed training took 9–33 training sessions (Mean = 20 ± S.D = 6.769) from habituation and TP1 to meeting the criterion at the end of TP3. The twenty dogs that completed cognitive bias tests gave 144 responses each to various cues over the three cognitive bias tests. One dog had data for only two cognitive bias tests as the equipment failed during the second test, resulting in no latency data for that test. The percentage of water tones responded to was calculated for the last two training sessions before testing commenced (n = 47 trials per dog) and the cognitive bias tests (n = 45 trials per dog) to examine possible effects of novelty on response rate. A Wilcoxon signed-rank test showed that the response rate for water tones calculated from 20 dogs before cognitive bias tests and those from the same 20 dogs during cognitive bias tests differed significantly (W = 210; p<0.001; r = 0.670).

**Figure 3:**
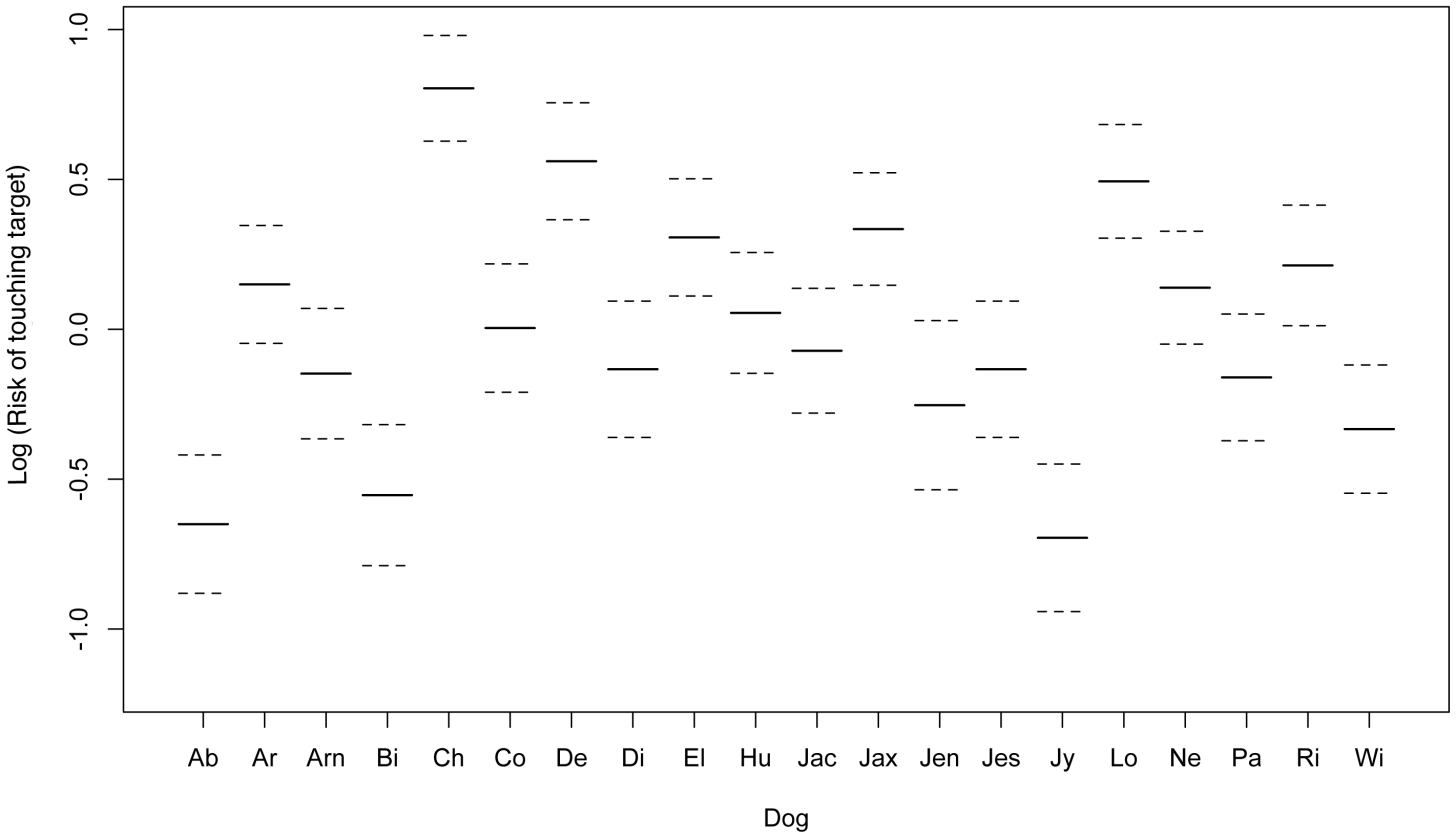
The risk of touching the target before the 10-second time out for all cues is shown on the y-axis in a log scale, and individual dogs (n = 20) are shown on the x-axis. Standard errors are shown with broken lines. Some dogs are much more likely to touch the target than others. For example, dogs “Ab”, “Bi” and “Jy” have a low likelihood of touching the target regardless of cue, and dogs “Ch”, “De” and “Lo” have a high likelihood of touching the target regardless of cue.

**Figure 4:**
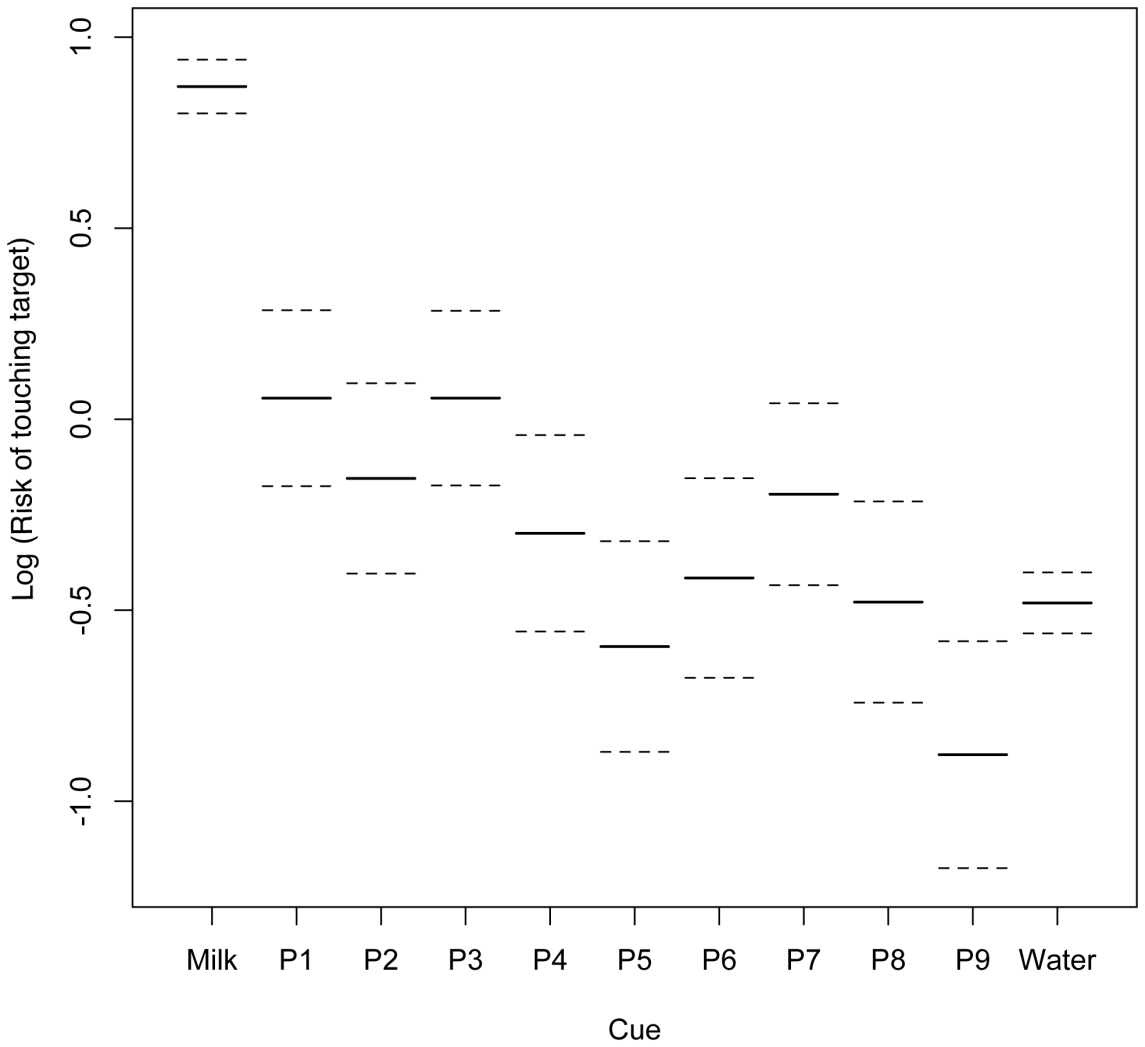
The log of the risk of all dogs (n = 20) touching the target before the 10-second time out for each cue shown on a log scale on the y-axis with the cues on the x-axis. Standard errors shown with broken lines. Risk is high for the milk tone, showing all dogs were highly likely to touch the target after the milk tone. The lowest risk was for P9, the probe most like water. This shows dogs were unlikely to touch the target after the P9 probe cue. P5, the most ambiguous cue, also showed a low risk of dogs touching the target after this cue.

The Cox's proportional hazards model showed that there was a significant effect of Dog (DF = 18.57, LRT = 261.86, P<0.001) and Cue (DF = 10.19, LRT = 616.9, P<0.001) as well as test number (DF = 2.0, LRT = 16.45, P<0.001) on latency and the risk of the dog touching the target within the 10-second window. “Risk” here is very similar to “likelihood”, but does not share the same statistical meaning. It may be considered the probability of an individual touching the target within the 10-second window while considering time in many small intervals. A summary of the terms included in the final model is shown in **Table 6**. Protocol did not have a significant effect on latency and the risk of touching the target in the survival analysis, and nor did the dog's background, and both terms were excluded from the model. The log of the risk of each dog touching the target within the 10-second window is shown in **Figure 3**. This shows that some dogs are far more likely to touch the target after any tone than others. **Figure 4** shows how the risk of dogs touching the target differs between cues. There was no significant difference in risk of touching the target between the first and second cognitive bias tests, but there was a significant decrease in the risk of dogs touching the target in the third test compared to the first, indicating that dogs were significantly less likely to touch the target in the third test.

**Table 6:** The statistical output of the final Cox Proportional Hazards regression model.

Term	Regression Coefficient	Standard Error	DF	p-value
Cue P1	−0.816	0.123	1	<0.001
Cue P2	−1.026	0.133	1	<0.001
Cue P3	−0.816	0.123	1	<0.001
Cue P4	−1.170	0.137	1	<0.001
Cue P5	−1.466	0.147	1	<0.001
Cue P6	−1.287	0.139	1	<0.001
Cue P7	−1.107	0.128	1	<0.001
Cue P8	−1.350	0.141	1	<0.001
Cue P9	−1.749	0.158	1	<0.001
Cue Water	−1.352	0.064	1	<0.001
Test 2	−0.007	0.059	1	0.91
Test 3	−0.213	0.059	1	<0.001
Frailty (Dog)			18.6	<0.001

Includes all dogs that complete the cognitive bias testing (n = 20). These data describe the difference between the latency of dogs touching the target after the milk tone (reference condition) to each probe tone (CueP1–CueP9) and the water tone (Cue Water). Negative regression coefficients show a reduction in the likelihood of reaching a certain event, in this case, touching the target. Thus, the likelihood of touching the target is significantly less after probe and water tones than after milk tones. The risk of touching the target was not significantly different between test 1 and 2, but was significantly less in test 3 than test 1, indicating a reduced likelihood of touching the target over successive tests. The frailty term (“Dog”) refers to the dog being tested, which is treated in this model as a random effect due to repeated measures on each dog. The term “Dog” also had a significant effect on likelihood of touching the target, meaning that individuals varied significantly in their latency to touch the target.

Response latency graphs were prepared for each dog that completed the cognitive bias tests. Graphs include the average latency for each tone, the standard deviation for each tone, and the probability of latency longer than the average latency for each tone. Variance scores for each dog that completed the cognitive bias tests are shown in **Table 7**.

**Table 7:** Details of dogs in the study that completed cognitive bias testing.

Dog	Source	Breed	Sex	Sessions to CBT	% milk responses at testing	% water responses at testing	Variance score
De	Public	Labrador retriever	M	13	100	89	6.47
El	Public	Labrador retriever	F	14	94	67	5.78
Ch	ADA	Labrador Mix	M	14	96	73	5.27
Ri	ADA	Labrador Mix	M	15	76	46	5.11
Co	ADA	Golden retriever	M	17	100	50	4.41
Ar	Public	Pug X Schnauzer	M	24	100	50	3.95
Arn	Security	German shepherd dog	M	33	90	60	3.71
Di	Public	Rhodesian ridgeback	M	16	75	33	3.51
Jes	Public	Border collie	F	15	96	60	3.51
Lo	Public	Labrador retriever	F	14	98	91	3.04
Pa	Security	English springer spaniel	F	24	90	50	2.8
Jen	Public	Border collie	F	28	94	58	2.69
Jax	ADA	Labrador retriever	M	20	100	82	2.05
Jac	Public	Australian cattle dog	M	17	98	77	1.47
Wi	ADA	Golden retriever	F	27	94	93	High latencies
Jy	Security	English springer spaniel	F	28	83	65	High latencies
Bi	ADA	Labrador retriever	M	18	70	36	High latencies
Ab	Public	Golden retriever	F	30	75	17	High latencies
Hu	ADA	Labrador retriever	M	19	96	64	High response rate
Ne	ADA	Labrador retriever	F	9	96	83	High response rate

Table shows source of dog, breed, sex, sessions to learning the discrimination task, percentage of each tone responded to in the two training sessions used to calculate a significant difference in latency prior to testing, and variance score computed from latency and standard deviation data related to unreinforced probe tones. High variance score indicates optimism. Variance score could not be calculated for some dogs as their data lacked a tipping point, a necessary attribute for this calculation. On occasions (n = 4), no tipping point could be determined due to overall high latencies such that average latency could not increase by 100% and still be within the 10-second response window. These dogs were categorised pessimistic. Two dogs displayed high response rates across all cues so that average latency did not increase by 100% between two adjacent cues.

**Figure 5:**
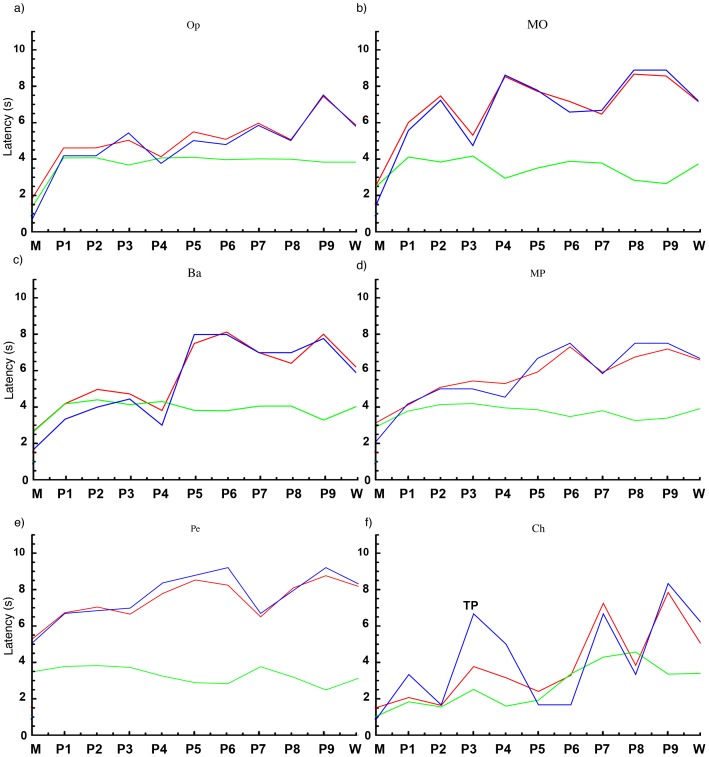
A series of graphs showing average latency (red), standard deviation (green) and log (probability of a slower than average response) (blue). In all graphs cue is on the x-axis, with probes arranged in a scale from closest to milk to closest to water. Latency in seconds is shown on the y-axis. Graph a) shows the pooled responses of dogs (n = 4), categorised as optimistic (1 on the rating scale in Table 8), characterised by standard deviation approaching the mean latency and average latency higher than the probability of a slower than average response. Graph b) shows the pooled responses of dogs (n = 4), categorised as moderately optimistic (2 on the rating scale). Standard deviation is lower, but the pattern of average latency is similar to that of optimistic dogs. Graph c) shows pooled responses of dogs (n = 3), categorised as balanced (3 on rating scale). Characteristics are similar to those in the moderately optimistic graph. Graph d) shows pooled responses of dogs (n = 3), categorised as moderately pessimistic (4 on rating scale). Average latency tends to be higher than in other graphs. Graph e) shows the pooled responses of dogs (n = 4), categorised as pessimistic, typified by high initial latencies and low standard deviation. Graph f) shows the responses of a single dog, characterised as optimistic. Tipping point can be seen where average latency increases by 100% or more, indicated by “TP”. Standard deviation approaches mean latency and probability of faster than average response remains high for much of the graph.

Variance scores were used to place dogs in optimism categories. Results from dogs with high variance scores, standard deviation approaching mean latencies, and average latencies higher than the probability of slower than average responses were pooled to show a typical graph for optimistic dogs (variance score >5). Results from dogs with moderate to moderately low variability in latencies, and moderate to high probabilities of slower than average responses were pooled to show a typical graph for moderately optimistic (variance score >3.5 and <5), balanced (variance score >2 and <3.5) and moderately pessimistic (variance score <2) dogs. The dogs with high latencies precluding variability scores and low standard deviation and high probabilities of slower than average responses were pooled to show a typical graph for dogs that were pessimistic. These graphs are shown in **Figure 5** alongside a graph from an optimistic dog to allow a comparison between the individual dog and the optimism category they were assigned to.

All dogs that completed cognitive bias testing had at least one optimism rating from an owner or trainer, and 18 of the 20 dogs had two or more ratings. There were not enough data to perform an inter-rater agreement analysis on ratings. A Spearman's rank correlation was performed on a mean of the trainer and owner ratings (n = 43) with the behavioural data. The results indicate a weak relationship that was not significant (r_s_ = 0.382, p = 0.118). Rater results are shown in **Table 8**. There was a tendency for owners and trainers to over-estimate the optimism of dogs belonging to pessimistic, moderately pessimistic, and balanced groups, and to under-estimate the optimism of dogs in moderately optimistic and optimistic groups.

**Table 8:** Subjective ratings of dog optimism from owners (n = 11) and trainers (n = 11).

Dog	Data rating	Owner 1	Owner 2	Trainer 1
De	**1**			1
El	**1**			3
Ar	**2**	1		1
Di	**2**			5
Jes	**3**	1	1	
Lo	**3**	1	3	1
Jen	**4**	5	3	
Jac	**4**	1	1	2
Ab	**5**	4	3	3

	Data rating	Trainer 1	Trainer 2	Trainer 3
Ch	**1**	2	1	
Ri	**1**	2	1	1
Co	**2**	2	2	
Arn	**2**	2	3	
Pa	**3**	1	2	
Jax	**4**	1	5	2
Wi	**5**	3	3	4
Jy	**5**	2	1	
Bi	**5**	3	3	3
Hu	**NA**	2	5	5
Ne	**NA**	1	1	

Data rating refers to optimism category assigned based on the dog's variance score. See Table 3 for descriptions of ratings. Ratings are an ordinal scale ranging from 1 = optimistic to 5 = pessimistic.

## Discussion

Latency to touch the target differed significantly between probes, with dogs being, on average, slower to touch the target as probes became more similar to the water tone. This supports the prediction that dogs would respond differentially to signals and that this may correspond to their expectations of positive and negative outcomes. The differing responses between dogs in this study suggest probes are interpreted differently at an individual dog level. While this seems to support the hypothesis that judgement bias exists in dogs and can be measured objectively, it is unclear how much the differences in responses between dogs can be attributed to affective state. Cognitive biases in humans are sensitive to both short-term changes in an individual's level of anxiety (state anxiety) and long-term, individual difference in an individual's tendency to experience anxiety (trait anxiety, dispositional optimism) [29]. There is evidence in animals that some individuals may be inherently more pessimistic than others, for example, stereotyping starlings and macaques are more pessimistic than non-stereotyping or reduced stereotyping conspecifics [13], [21], and dogs that show indications of separation-related distress are more pessimistic than dogs that do not [23]. Dogs from Assistance Dogs Australia and security dogs in this study shared the same training and trainers, and the same care and management practices with all the other dogs from their facility, providing largely standardised conditions within each group. Differences in responses between dogs housed at the same facilities may represent a fundamental difference in individual dogs' ability to cope with challenging environments, or an inherent tendency towards optimism or pessimism akin to the trait anxiety described above.

Dogs in this study had a higher risk of touching the target after the water tone than some probe tones. This has not been observed in other judgement bias studies in dogs. There were many probe tones presented during testing. It is possible dogs responded to familiar tones more readily than unfamiliar probe tones, and sometimes made errors in discrimination as a result. This is unlikely to be a case of the mere exposure effect, whereby stimuli become preferred simply through repeated exposures, as this is associated with neutral stimuli and positive affect [30]. Dogs have been shown to prefer novel stimuli over negative stimuli [31] but it is possible, given the low cost of an error in discrimination in this task, that neophobia may to some degree overcome the avoidance of errors. An examination of whether particular dogs were responsible for the overall elevated response rate to water tones and whether these dogs were the more pessimistic individuals may offer support for a role of neophobia in these results. Percentage of water tones responded to in the training sessions immediately prior to testing are presented in Table 7, and show large variation in response rate to water tones across all optimism scores. The percentage of water tones responded to differed significantly between these training sessions and cognitive bias tests but it declined over time, suggesting that neophobia does not play a role in the response rate to water. Future explorations into the role of motivation on cognitive bias results would likely be very beneficial. Finally, reducing the number of probes or the frequency with which they are presented and comparing results would be a worthwhile exercise to establish whether a large number of probes affects discrimination between the milk and water tone. It would also help to establish the ideal number of probes.

The high exclusion rate was problematic in this study, and may result in a skewed representation of base level optimism in dog populations if used in its current form. Further refinements of the design and program would likely improve this. Using food rather than a liquid reward may improve motivation to interact with the apparatus, and making the transition between training phases more gentle, such as with slower reductions in reinforcement rate, may also lower the exclusion rate. A version of this apparatus that operated completely automatically and delivered a large portion of a dog's daily food allowance through interaction with it is anticipated to solve many of the exclusion rate problems.

The exclusion rates differed between groups. It is unclear from the data collected why security dogs had a higher exclusion rate than the other two groups. Their training differed considerably from that of the companion dogs, all of whom were recruited through a training school with an emphasis on positive reinforcement, and the assistance dogs, who were being trained for much calmer and steadier responses than the security dogs. Many of the security dogs were excluded early in the training and typically took twice as many sessions for them to progress to TP2, if they did at all, than it did for dogs in the other two groups. This may hint at difficulties with reward saliency.

The test number had a significant effect on latency and risk of touching the target. This was analysed to search for a learning effect, which would manifest in dogs responding to fewer probes over time as they learn that probes are not reinforced. This effect has been documented in sheep [32] and starlings [33], but despite being searched for in dogs, has not been identified [23]. There was no significant difference between the first and second tests, but there was a significant decline in latency and risk of touching the target in the third test compared to the first. It is possible this effect was not found before in dogs because the method used by Mendl et al. [23] required fewer trials (21–61 as opposed to at least 9 sessions of 48 trials each in this study) with fewer probes (4 vs 9 in this study), thus not giving dogs (n = 24) the opportunity to learn that probes are unreinforced. A refinement of the methodology presented here by reducing the number of probes may aid in reducing the test effect. However, reducing the number of probes may also reduce the power of detecting fine scale differences in optimism and pessimism between dogs. It was beyond the scope of this study to test the optimal number of probes to present, and this is part of the cognitive bias methodology that has not yet been systematically investigated. The data presented here suggest steps should be taken in future studies to avoid a possible effect of test number.

The statistical model detects broad patterns and differences in the data, but does not provide the means to interpret the data of individual dogs. We have taken a novel approach in interpreting the data of individual dogs using a simple mathematical model in addition to the statistical model. This is a preliminary measure that ideally will be honed with additional data in the future. Examining patterns in mean response latency reveals clear tipping points (see Table 4 for definition) in most dogs, showing a specific tone where dogs' average latency is longer or the response rate drops sharply from the previous tone. The location of tipping points on the scale between the learned positive and negative tones varied between dogs. This may indicate differences in interpretation of ambiguous tones, suggesting differing judgement biases. An alternative interpretation is that differences in tipping point may reflect learning differences in cue discrimination. Discrimination was assumed to have occurred when responses to milk tones were significantly faster than responses to water tones for two of three consecutive sessions. Despite this statistical approach to the criterion for testing cognitive bias, it is possible some dogs had different error rates than others for the milk and water tones when their cognitive bias was tested, and this may have influenced their tipping point.

Examining the variability of responses after the tipping point is therefore likely to be most revealing of optimism as it does not depend on discrimination ability. The tipping point shows that dogs are discriminating between tones and supports the hypothesis that they are interpreting some ambiguous signals as signalling a positive outcome and some as signalling a negative outcome as well as pinpointing where that switch in interpretation occurs. Standard deviations that approach the mean latency coupled with lower probabilities of a latency longer than average after the tipping point suggests that the dog is responding to some probe tones that are, on average, provoking long latencies such as those associated with the water tone with short latencies akin to those associated with the milk tone. This may indicate that either the dog is interpreting a proportion of those probes after the tipping point as signalling a positive outcome or the dog is taking risks by responding to some ambiguous signals in case they are signalling a positive outcome. We propose that either interpretation is a stronger indication of optimism than the tipping point alone. Conversely, standard deviations lower than the average latency and high probabilities of longer latencies than average after the tipping point indicates the dog is responding to the majority of probes after the tipping point with long latencies or not touching the target at all. This suggests that the dog is either interpreting a greater proportion of probes after the tipping point as signalling a negative outcome or is not willing to risk touching the target in case the ambiguous signal indicated a negative outcome. We propose that either interpretation is a stronger indicator of pessimism than tipping point alone. Validation of this method was not possible in this study due to resource constraints, but remains a difficulty in cognitive bias studies on animals in general. Some studies have found evidence that physiological measures indicating heightened stress correlate with pessimism [17], [25]. However, a disconnect between cortisol concentration and judgement bias has been reported in sheep [15], and in some cases, both cortisol concentration and judgement bias have failed to differentiate between treatments [34], [35]. It is possible that taking into account typical baseline cortisol concentrations, typical cortisol responses and inherent optimism or pessimism in individuals may improve the sensitivity and efficacy of the method. Previous studies have shown tantalising potential in the use of judgement bias in assessing affective state in animals, but results may be confounded by factors such as individual motivation, reward and signal salience, and personality. Until such factors have been accounted for in judgement bias data, validity may prove elusive, results may vary, and comparisons between methods may be of limited use.

The variability score calculated from standard deviation and average latency at each probe after the tipping point gives a single measure of the conditions described in the previous paragraph and thus a possible surrogate for a single optimism score. This gives the opportunity to place dogs on a pessimistic-optimistic scale and compare their degree of optimism with that of other dogs. This represents a more detailed interpretation of judgement bias data than that presented in any other animal studies to date. It is anticipated this mathematical model can be improved on with more data that may allow a weighted algorithm taking into account tipping point and variability score differentially. One potential problem with the current optimism index is that it relies heavily on standard deviation with the assumption that, on average, responses after the tipping point are slow or there is no response at all. A dog with very short latencies may show a tipping point, yet also respond very quickly to many probe tones, in which case the standard deviation may be small and the resultant optimism score may be lower than it should be were it truly reflecting optimism for that dog. As such, including a measure of response rate in the anticipated algorithm may improve the accuracy of the optimism score.

There was no significant correlation between the optimism rating of owners and trainers and the data. Owners and trainers tended to label optimistic and moderately optimistic dogs as less optimistic than our empirical data suggested, but balanced, moderately pessimistic and pessimistic dogs as more optimistic than the data suggested. This may reflect the subset of the dog population that completed testing. There was also a difference between dogs in different populations that may alter the experiences of the owners and trainers with dogs in general. For example, the exclusion rate was very high in security dogs and of the three security dogs that did complete the tests none were in the optimistic group. Trainers working with such dogs are likely to label them relative to other dogs in that population, which may be skewed towards pessimism, leading to elevated optimism ratings, as indeed occurred in the two dogs that were categorised (according to the empirical data) as balanced and pessimistic.

There is growing empirical support for the use of judgement bias in objective assessment of affective state in animals [16], [17], [25]. The focus of this study was not on validating this method as a measure of affective state, and as such the dogs in this study were not subjected to any manipulations intended to alter their affective state, and no measures of affective state were attempted. Therefore, no conclusions can be drawn from this study regarding the efficacy of judgement bias in measuring affective state in dogs. However, the variation seen in responses from dogs even within the same facilities suggests that personality may play a role in judgement bias results that has not been quantified as yet. Further research in judgement bias in animals should address the possible impact of personality on test results and consider how this may confound future attempts to find a treatment effect in groups of animals assumed to be roughly equal in susceptibility to a given treatment.

Further research into the personality of dogs excluded from the study may reveal patterns in personality traits that may explain why some dogs were not able to complete the training. It is likely a certain level of optimism is necessary for dogs to persist with the self-directed training when reinforcement rates drop as the training progresses. The reinforcement rate was stepped down over three phases during training, which was adequate for many dogs, but may have been too fast or have included a drop between phases that was too large for other dogs. A study that found that rats were more sensitive to reward loss when their welfare was compromised [36] may help to explain why dogs failed to meet criteria during training. Although it is difficult to draw parallels between reward loss and a reduction in reinforcement rate, further research into the personality of those dogs being excluded due to extinction of the targeting behaviour may prove insightful.

## Conclusions

This study provides proof of concept for the use of a portable, automatic apparatus used to both train dogs and test their cognitive bias. It also lends support to the use of cognitive bias as a tool to objectively measure affective state in dogs. Further research into extinction curves and personality of dogs that were excluded from the study may reveal important information about the affective state of dogs that failed to respond adequately to early training. The addition of a descriptive mathematical model to interpret cognitive bias data offers distinct advantages over a purely statistical interpretation, but may require some development.
